# Immunohistochemical Expression of Epithelial Mesenchymal Transition Proteins in Squamous Cell Carcinoma of the Oral Cavity

**DOI:** 10.30699/IJP.20201.137498.2502

**Published:** 2021-07-06

**Authors:** Hamideh Kadeh, Shirin Saravani, Ebrahim Miri Moghaddam

**Affiliations:** 1 *Oral & Dental Disease Research Center, Department of Oral & Maxillofacial Pathology, School of Dentistry, Zahedan University of Medical Sciences, Zahedan, Iran *; 2 Department of Molecular Medicine, Cardiovascular Diseases Research Center, School of Medicine, Birjand University of Medical Sciences, Birjand, Iran

**Keywords:** E- cadherin, N-cadherin, Oral Cancer, Epithelial- Mesenchymal Transition (EMT)

## Abstract

**Background & Objective:**

Epithelial-Mesenchymal transition (EMT) is known to be a possible mechanism in tumor progression; however, there is insufficient evidence to support the contribution of this process in human cancers. The present study aimed to evaluate the expression of EMT markers in normal oral epithelium and oral squamous cell carcinoma and also correlates with some clinicopathological parameters.

**Methods:**

This study was conducted on 70 samples, including 20 cases of normal epithelium and 50 cases of Oral Squamous cell Carcinoma (OSCC). To examine the expression level of these proteins, immunohistochemical staining was performed for samples using E-cadherin and N-cadherin monoclonal antibodies.

**Results:**

Reduced expression of E-cadherin was observed in 74% of OSCC and 15% of normal epithelium samples; this difference was statistically significant (*P*˂0.000). With the progression of SCC from well towards poor differentiation, the E-cadherin expression decreased; however, this difference was not statistically significant (*P*=0.642). Normal epithelial specimens were negative for N-cadherin expression in 75% of cases, whereas OSCC specimens showed high expression of N-cadherin in 46% of cases, this difference was statistically significant (*P*=0.01). Although 62.5% of poorly differentiated OSCC showed high expression of N-cadherin, the difference between the histopathological grades was not significant (*P*=0.586). No significant relationship was found between markers expression and patient’s age, gender, and tumor location.

**Conclusion:**

This study showed that OSCC tissues showed high EMT phenotype (reduced E-cadherin expression and high expression of N-cadherin) compared to normal oral mucosa which may indicate the possible key role of EMT mechanism during oral carcinogenesis.

## Introduction

Oral Squamous Cell Carcinoma (OSCC), is the sixth most prevalent cancer in the world (1-4). More than 400,000 people die due to OSCC annually. The 5-year survival rate for patients with SCC is relatively weak and less than 60% (5-7). Poor prognosis of OSCC is due to its invasion into adjacent tissues and metastasis; therefore, identifying biological markers which can provide prognostic information of tumor is helpful (8-10).

The EMT is an early cellular process which is involved in tissue remodeling during development. In cancer, the EMT plays a critical role in tumor prog-ression to more aggressive phenotype (11). During the EMT process, polarized epithelial cells lose their polarity and cell-cell adhesion; they transform into mesenchymal cells and actually acquire the charac-teristics of invasion and migration.(12-14). One of the well-known processes in EMT is breakage in junctions between epithelial cells and reduction in the E-cadherin expression (epithelial cadherin), whereas cells clearly express high levels of N-cadherin (mesenchymal cadherin). The aforementioned process is called cadherin switching (12-15).

Oral epithelial cells are joined by tight cell-cell adhesion mediated by cadherins. The cadherins are a large family of widely regulated and conserved calcium-dependent membrane-binding proteins. The E-cadherin and N-cadherin are prominent members of the cadherin family (16, 17).

The transmembrane glycoprotein E-cadherin plays a vital role in maintaining the structural integrity, function of adhesion molecules and desmosomal junctions. In addition, it regulates various aspects of cellular behavior (e.g., growth, differentiation, polari-zation, and classification of epithelial cells). This gly-coprotein is primarily responsible for cell-cell adhesion in epithelial tissue, and it lack increased epithelial cell motility and local invasion ability, which is recognized as an EMT sign during tumor progression (13, 18).

The N-cadherin is a calcium-binding protein which has been found primarily in neural and striated muscle tissues. Increased expression of N-cadherin has been reported in tumors, such as leiomyoma, pheochromocytoma, adrenocortical carcinoma (19).

Although epithelial cells do not express N-cadherin, they may acquire it during the cadherin switching process. This process occurs during EMT and enhances cellular motility and their invasive properties (13, 20).

Several studies and experimental models have indicated that the EMT is an important mechanism in malignant phenotype and metastasis in cancers. However, its role in human cancer, including head and neck SCC is controversial (21-23). 

Since studies on EMT and potential relation with histopathological grading in Oral cancer are sparse (24), this study aims to evaluate the expression of EMT- associated proteins E-cadherin and N-cadherin in OSCC.

## Material and Methods

Case Selection

Following the approval of this study by local Ethics Committee (7479) we analyzed 70 specimens consisting of 50 OSCC and 20 normal epithelia obtained from department of oral pathology in Zahedan Dental School, Iran, by census sampling method between 2005 and 2019. This was a retrospective study using previously diagnosed samples. All H&E slides were reviewed for confirmation of diagnosis and classification of the OSCC samples as well differentiated, moderately differentiated and poorly differentiated, according to International Histological Classification of Tumors (25). Demographic and clinicopathological data of the samples were extracted from patient’s records.

 For control group, the normal epithelium without inflammation was obtained from the gingiva tissue (patients who had undergone crown lengthening surgery and had no history of head and neck carcino-mas). All samples with sufficient tissue were included for immunohistochemical analysis. 

Immunohistochemistry

For IHC staining, paraffin- embedded samples were segmented into 4 µm sections, the samples were deparaffinized with xylene and rehydrated with 100%, 90% and 80% alcohols solution. Antigen retrieval was performed by microwave heating for 30 min on Tris buffer (pH = 7.6). Then, the sections were incubated for 1 h at room temperature with primary mousem-onoclonal anti-human antibody N- Cadherin Clone 6044777 (Novocastra, United Kingdom), E- Cadherin Clone 36B5 (Novocastra, United Kingdom) according to the manufacturer’s instructions. Then, sections were washed with PBS at room temperature, and secondary antibody was applied. The immune complexes were incubated with streptavidin peroxidase (Novo Link Polymer Detection system). The immune reaction was developed with diaminobenzidine and counterstained with Mayer’s hematoxylin. Finally, the sections were dehydrated in alcohol and cleared in xylene, and slides were mounted in Permount. In negative controls primary antibody was omitted. The samples of Breast cancer tissue and normal testis were used as positive control for E-cadherin and N-cadherin respectively.

The result of immunohistochemical staining was evaluated by a pathologist under a light microscope (Nikon, Type2, Tokyo, Japan). Immunostaining was assessed in 10 high-power fields (HPF) at a magnification of 400, and scored as described in other studies (26, 27). For N-cadherin the samples were divided into low expression (membranous and cyto-plasmic immunostaining in less than 20% of the epithelial tumor cells) and high expression (mem-branous and cytoplasmic immunostaining in 20% or more of the epithelial tumor cells). E-cadherin was defined as preserved (membranous immunostaining in more than 80% of the epithelial tumor cells) and reduced (membranous immunostaining in 80% or less of the epithelial tumor cells). 

 Statistical Analysis

Data analysis was performed using SPSS 21(SPSS Inc, Chicago, IL., USA). The relationship between the groups was assessed by Mann-Whitney and Kruskal Wallis tests. P-value less than 0.05 was considered statistically significant.

## Results

In the current study, 70 specimens were examined (including 50 cases of OSCC with mean age of 57.1±15.18 years and 20 cases of the normal epithelium as the control group with mean age of 33.45±5.34 years). Table 1 shows the demographic data of OSCC samples.

**Table 1:** Demographic data of OSCC samples

Clinical data		Frequency N (%)
Gender	Male	19(38)
	Female	31(62)
Tumor site	tongue	11(22)
	Mandibular gingiva	14(28)
	Maxillary gingiva	3(6)
	Buccal mucosa	10(20)
	lip	5(10)
	intraosseous	1(2)
	others	6(12)

**Fig 1:**
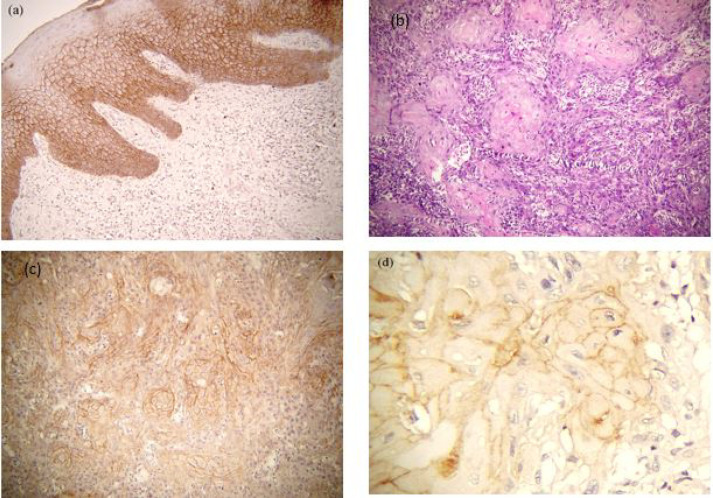
(a) E- cadherin expression in normal oral epithelium (×100). (b) H & E section of moderately differentiated OSCC. (c) Reduced expression of E- Cadherin in OSCC in same H &E microscopic field (×100). (d) Reduced expression of E- Cadherin in OSCC (×400)

In normal epithelium, basal and suprabasal cells showed the highest amount of membrane staining in relation to E-cadherin, and gradually decreased in cells that had undergone to keratinization (Figure 1a).

About 74% of SCC samples showed reduced expression of E-cadherin but in normal epithelium only 15% of cases showed reduced expression of E-cadherin and according to the Mann-Whitney test this difference was significant (*P*<0.000). The results are expressed in Table 2 and Figure 1.

**Table 2:** Expression of E-Cadherin and N-cadherin in OSCC and Normal epithelial cells

Group	No.	E-Cadherin		P-value	N-Cadherin			P-value	Total
		Preserved	Reduced		Negative	Low	High		
Normal	20	17(85%)	3(15%)	<0.000*	15(75%)	5(25%)	0	0.01^*^	20(100%)
OSCC	50	13(26%)	37(74%)		25(50%)	2(4%)	23(46%)		50(100%)

*Significant- Mann-Whitney test

In addition, the results of the present study showed reduction in E-cadherin expression with progression of SCC from well to poor differentiation; however, this difference was not statistically significant (*P*=0.642) (Table 3).

The expression of E-cadherin was not correlated with patient’s gender (*P*=0.899), age (*P*=0.790) and location of tumor (*P*=0.311).

**Fig 2:**
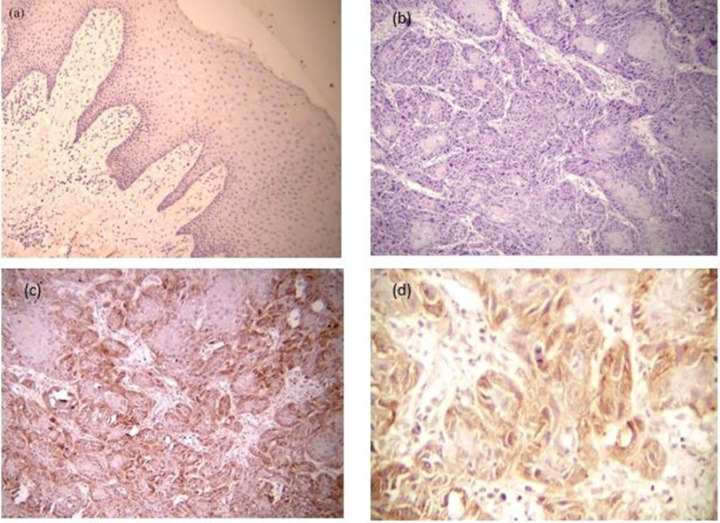
(a) Negative expression of N-cadherin in normal oral epithelium (×100). (b) H & E section of moderately differentiated OSCC (×100). (C) High expression of N-cadherin in OSCC in same H&E microscopic field (×100). (d) High expression of N-cadherin in OSCC (×400)

Regarding the expression of N-cadherin, normal epithelial specimens were negative in 75% of cases and did not express this protein and only 5(15%) cases showed low expression of N-cadherin. Whereas in the SCC group, 50% of the samples showed no expression of N-cadherin and 46% showed high expression of N-cadherin and according to Mann-Whitney test the difference between the groups was significant (*P*=0.01) which are shown in Table 2 and Figure 2.

The majority of well differentiated cases (60.9%) did not express N-cadherin. Moreover, moderately, and poorly differentiated of OSCC samples showed 52.6% and 62.5% high expression of N-cadherin, respectively; however, these differences were not significant (*P*=0.586) (Table 3).

No relationship was observed between N-cadherin expression and patient’s gender (*P*=0.055), age (*P*=0.511) and location of tumor (*P*=0.471).

Also, as shown in Table 4, out of 50 SCC cases, 26% (n=13) showed cadherin switching (reduction in the E-cadherin expression and the subsequent gain of N-cadherin expression in the same cell population).

**Table 3:** Expression of E-Cadherin and N-cadherin in different histopathological grades of OSCC

Group	No.	E-Cadherin		P-value	N-Cadherin			P-value	Total
		Preserved	Reduced		Negative	Low	High		
WDSCC	23	7(30.4%)	16(69.6%)	0.642^*^	14(60.9%)	1(4.3%)	8(34.8)	0.586^*^	23(100%)
MDSCC	19	5(26.3%)	14(73.7%)		9(47.4%)	0	10(52.6%)		19(100%)
PDSCC	8	1(12.5%)	7(87.5%)		2(25%)	1(12.5%)	5(62.5%)		8(100%)

*Not significant- Kruskal Wallis test

WDSCC: Well Differentiated SCC, MDSCC: Moderately Differentiated SCC, PDSCC: Poorly Differentiated SCC

**Table 4:** Cadherin Switching in different histopathological grades of OSCC

Group	No.	Cadhering switching		Total
		Positive	Negative	
WDSCC	23	4(17.4%)	19(82.6%)	23(100%)
MDSCC	19	6(31.6%)	13(68.4%)	19(100%)
PDSCC	8	3(37.5%)	5(62.5%)	8(100%)

WDSCC: Well Differentiated SCC, MDSCC: Moderately Differentiated SCC, PDSCC: Poorly Differentiated SCC

## Discussion

The EMT is defined as the switching of polarized epithelial cells to the fibroblastoid migratory phenotype which occurs in many evolutionary processes and during tumor metastasis; however, the precise role of this process in tumor development or progression is still a debating issue (19, 28).

In the present study, the expression of E-cadherin and N-cadherin proteins in OSCC and normal epithelium samples were examined. Based on the current findings, the OSCC samples showed a reduced expression of E-cadherin, compared to the control group. In addition, although 75% of the control group samples did not express N-cadherin, 46% of SCC samples showed high expression of N-cadherin and this difference was significant. The obtained results denoted that EMT plays a possible key role in the development and evolution of OSCC.

Furthermore, in the present study, with the prog-ression of SCC from well towards poor differentiation, reduction in E-cadherin protein expression and high expression of N-cadherin was observed; however, these differences were not statistically significant due to the low sample size and inappropriate distribution of the samples selected from various histopathological grades.

Similar to the present study, Guo* et al.* (29) showed that from normal oral mucosa to OSCC and with the progression of tumor, E-cadherin expression decreased and N-cadherin expression increased (both human and animal models). Also, they revealed that E-cadherin expression in metastatic lymph nodes increased again. It is well known that switching from E-cadherin to N-cadherin may be due to the loss of adhesion between cells, which causes the tumor cells to separate from the primary tumor, migrate and eventually metastasize. According to the aforementioned study, when cancer cells reach metastatic target tissue, E-cadherin expression occurs again resulting in colonization of cancer cells in invasive tissues. This condition is probably a dynamic adjustment of the proteins expression with micro-environmental changes and the E-cadherin expression is time- and space-dependent. 

Pyo* et al.* (30) found that reduced expression of E-cadherin and increased expression of N-cadherin are associated with OSCC and suggested that cadherin switching plays an important role in the development of OSCC.

Angadi* et al.* (24) reported a high level of EMT phenotype (decreased E-cadherin and ß-catenin expression and increased N-cadherin expression) in OSCC tissues.

Zidar* et al.* (23) showed that decreased focal E-cadherin expression was observed in a very few cases of oral SCC and most cases showed fully preserved E-cadherin expression. In addition, N-cadherin expression was not observed in any of the OSCC cases. They were not found, any evidence that EMT contributes to SCC progression and metastasis, which is contrary to our study. Zidar* et al. *reported that the case-selection was one reason for their findings. It means that, all patients at the early stages of SCC were moderate differentiation; therefore, the potential role of EMT in progression and metastasis cannot be ignored at higher OSCC stages or its poor differentiation. 

Ozaki-Honda* et al.* (31) investigated the expression of E-cadherin and N-cadherin in OSCC cells in two areas of tumor nest and invasive front. Cells expressed N-cadherin and E-cadherin in both regions; however, only N-cadherin expression in both regions was associated with the patient prognosis and was reported to be a helpful marker in the diagnosis of small tumor satellite and OSCC cells with the EMT.

In another study performed by Rai* et al. *(12), it was shown that with progression of tumor from low to high histopathological grade, the expression of N-cadherin protein increased. Accordingly, N-cadherin in different grades of SCC can be useful in predicting tumor progression.

Costa* et al.* (13) studied the expression of EMT markers (E-cadherin, N-cadherin and vimentin) in OSCC. Decreasing in E-cadherin expression was observed in 75% of OSCC samples in the invasion front region; it was also related to histological invasiveness. All cases of OSCC were negative for N-cadherin and 30% of OSCC were positive for vimentin, there was no correlation between the expression of each protein in IF with the tumor stage or nodal status. Also, no correlation was found between E-cadherin and N-cadherin expression (13).

The E-cadherin is the major adhesion molecule in epithelial tissues, it is required to establish and maintain the integrity of the epithelial structure. Several mechanisms have been proposed to inactivate E-cadherin protein in human malignant tumors. Since the lack of E-cadherin increases the mobility of epithelial cells, an EMT marker is considered during the metastatic invasion. 

Several studies have shown that although cadherin-switching is required for increasing the motility, it is not necessary for EMT-related morphological changes (19).

Hashimoto* et al.* (16) also reported reduced expression of E-cadherin in OSCC, this decrease was related to tumor differentiation; however, N-cadherin positivity was very limited and did not correlate with clinicopathologic parameters. Moreover, in this study, mouse tongue tumors xenotransplanted oral SCC cell lines expressed both N-cadherin and E-cadherin in vitro; however, in vivo conditions, N-cadherin expression were negligible, despite E-cadherin depletion. Therefore, reduction in the E-cadherin expression rather than cadherin-switching contributes to the OSCC progression. Also, the surrounding environment of carcinoma cells affect cadherin expression largely (16). 

Conversely, in a study performed by Domenico* et al.* (19), the expression of N-cadherin in neoplastic tissues was higher than normal and it was correlated with histopathological grade and stage of the patients. In addition, it was found that with increasing in the N-cadherin expression, OSCCs show poor prognosis and become more prone to metastasis. Lawson* et al.* (32) also reported increase of invasiveness in N-cadherin expressing cells of OSCC. 

Another study demonstrated that, the 5-year metastatic risk in patients with low expression of E-cadherin was more than patients with high expression of E-cadherin (81% versus 19%). Also, all Head and Neck SCC patients with EMT phenotype (low expression of E-cadherin and high expression of vimentin) had higher distant metastasis formation (33). 

Kong* et al.* in their study examined EMT related markers (CDH1, LAMC2, SNAI1/2, TWIST1, ZEB1 and ZEB2) in OSCC. In this study reduced expression of CDH1 (E-cadherin), overexpression of LAMC2, SnaiI1/2 and TWIST1 were observed. Loss of CDH1 was correlated with Broder’s grading significantly, while diffused LAMC2 was correlated with non-cohesive pattern of invasion similarly. Remarkably, co-expression of TWIST1 and ZEB2 was correlated with poorer overall survival in OSCC, particularly in patients with no lymph node metastasis (34).

The N-cadherin supports the systemic spread of tumor cells by enabling their association with stroma and endothelium in distant locations. It has been suggested that the interaction of cadherins with other cell-surface proteins (e.g., growth factor receptors) is the probable reason that E-cadherin and N-cadherin exhibit different phenotypes and behaviors in similar cellular contexts. (19).

Therefore, further studies on cadherin regulation by cellular environments are needed to understand the mechanism of OSCC progression. 

## Conclusion

According to the results of the present study, reduced expression of E-cadherin and high expression of N-cadherin are probable factors involved in the progression of normal mucosa toward SCC. Further-more, they are valuable markers in the EMT process during tumor progression.
